# *Compassionate Care—Going the Extra Mile*: Sex Trafficking Survivors’ Recommendations for Healthcare Best Practices

**DOI:** 10.3390/sexes2010003

**Published:** 2021-01-06

**Authors:** Arduizur C. Richie-Zavaleta, Augusta M. Villanueva, Lauren M. Homicile, Lianne A. Urada

**Affiliations:** 1 Department of Public Health, College of Graduate & Professional Studies, University of New England, Portland, ME 04005, USA; 2 Department of Community Health and Prevention, Dornsife School of Public Health, Drexel University, Philadelphia, PA 19104, USA; 3 School of Social Work, College of Health and Human Services, San Diego State University, San Diego, CA 92182, USA; 4 School of Medicine, University of California San Diego, 9500 Gilman Dr, La Jolla, CA 92093, USA

**Keywords:** human trafficking, empathy, identification recommendations, medicine, resources

## Abstract

Human Trafficking (HT) persists in the US, despite multi-level measures designed to mitigate its societal costs. HT instruction for healthcare providers is growing, but there is a dearth of resources and training presenting obstacles for victims accessing suitable healthcare services. Voices of survivors are also scant in the literature, despite the fact that their recommendations would appear essential when designing best practices. This study aimed to methodically gather recommendations from sex trafficking (ST) survivors who sought medical care during their victimization. An exploratory concurrent mixed-methods design was used, and semi-structured interviews (*N* = 22) were conducted between March 2016 and March 2017, in San Diego, CA, and Philadelphia, PA. Data were analyzed through a coding system to identify meaningful analytical themes. Study participants were recruited through survivor-centered organizations, and their identification was kept anonymous and confidential. Findings included three main themes: (A) Red Flags; (B) supportive healthcare practices; and (C) resources for ST-patient study participants’ recommendations aimed to improve healthcare practice in response to their medical needs in a compassionate and caring manner, with trust building, rapport, and an opportunity to instill hope among ST-patients. Implementing *Compassionate Care* approaches when caring for ST-patients could positively impact patient–provider interactions, while creating opportunities for intervention.

## 1. Introduction

The *Trafficking Victims Protection Act* (TVPA) was enacted in 2000 as one of the most significant federal laws in the 21st century. This law defined and criminalized human trafficking (HT) with the goal of eradicating the crime and protecting its victims [1]. In the last two decades, society at-large has become increasingly aware of this crime and the atrocities victims suffer under the oppression of their traffickers and the ramifications it has on their health outcomes [2-6]. Civil society, as well as governmental and nongovernmental agencies, have fought against this crime by updating the initial law and creating subsequent laws at the state level [7]. These efforts have also included educating the public and creating protocols to identify victims in a diversity of sectors, as well as evaluation of anti-trafficking efforts nationally and internationally [6,8,9]. However, there continues to be gaps in the efforts of prevention, intervention, and legislation at multiple levels within the US and abroad; especially among adult victims [7,10-12]. This research focuses on sex trafficking (ST) survivors’ recommendations to healthcare providers regarding best practices based on their experiences and interactions with a range of diverse healthcare settings and providers. Given the complexities in the victimization and identification of ST-patients; the framework of care here presented—*Compassionate Care*—could potentially assist healthcare providers to identify, support and assist ST-patients. Although ST-patients may overlap with other vulnerable populations in the healthcare setting, it is imperative to understand that ST-patients require specific assessment and treatment in order to meet their medical and other needs. Failing to recognize their victimization leads to inadequately providing them with the right resources to support their transition to survivorship [4-7,9]. What is worse, if the healthcare setting is not equipped to support such a population after identification, this could place the ST-patient in a greater danger.

HT continues to be an egregious crime committed against the most vulnerable of victims, particularly women and children [13]. Within HT there are different types of exploitation. TVPA defines ST when there is “recruitment, harboring, transportation, provision, or obtaining of a person for the purpose of a commercial sex act” [1]. When this type of crime is committed against a minor, the absence of force, fraud or coercion does not disregard trafficking as a crime, and it is considered a severe form of trafficking [1,13]. Other *severe* forms of trafficking occur when a person is entrapped into “involuntary servitude, peonage, debt bondage, or slavery” [1]. In practices, victims of ST are subject to strategic deception, confinement, reidentification, and exposure to violence, danger, and drugs. These strategies are used by traffickers in order to manipulate and control their victims. Therefore, trafficked victims, whether in the US or abroad, do not have the freedom to simply leave their perpetrators [1,13,14].

### 1.1. Marginalized Groups with Higher Risks for Sex Trafficking

In society, there are several marginalized groups who are at a higher risk of becoming trafficked. Their vulnerabilities originate from a diversity of needs—physical, emotional, social, and financial among others. Additionally, experiences of past trauma and abuse also contribute to becoming a trafficked victim. The literature has identified several characteristics among survivors that speak to the susceptibilities of becoming an easier target for sex traffickers. These characteristics include the following: (a) homelessness or runaway; (b) experiences of abuse or trauma—physical, emotional, or sexual; (c) being part of juvenile correction or child welfare systems; (d) between the ages of 12 and 14; and (e) member of a non-conformant gender group—LGTBQ; or see References [3,6,13-20]. In all, addressing the wide range of risk factors could lead to also reducing the number of victims of HT.

### 1.2. Prevalence and Its Challenges

Under TVPA (2000), HT crimes are classified as the fraudulent act of perpetrators exploiting victims into labor or ST or a combination of both [21]. The violence inflicted on the victims of these industries is grave [18]. Victims rescued and restored into survivorship have been diagnosed with acute physical and sexual trauma and adverse health effects including mental illness, substance abuse, sexually transmitted diseases, HIV infection, pregnancy, and abortion-related complications [3,22]. The estimates of victims of HT are far from reliable due to inconsistencies in the national systematic approach to estimate this criminal activity [11]. However, some international efforts have led to some estimates that may begin to paint a picture of how many victims exist across the globe. The Global Slavery Index estimates that there are around 403,000 children, women, and men in the US who are forced into an environment of violence and exploitation [23]. The Polaris-operated US National Trafficking Hotline has intervened in HT incidents involving 23,078 individual survivors: nearly 5859 potential traffickers, and 1905 trafficking businesses. Even though copious literature has quantified data regarding the severe magnitude of this global phenomenon of bondage, there is still a considerable number of cases that have gone unnoticed [23]. The true extent of its scope and magnitude are unknown.

### 1.3. Points of Entry—Opportunities for Intervention

To appropriately address HT—both sex and labor, it is vital that medical providers, social services professionals, and trained law enforcement agents, follow effective frameworks and safe protocols to perform appropriate measures in order to assist in the transition from victimization to survivorship when the opportunity arrives [14]. The literature speaks of collaborative models that work best in addressing ST-patients in the context of healthcare settings. Both coordination of services and needed resources to move beyond victimization are essential [6,10,24,25]. In the context of healthcare settings, frontline staff, clinicians, and social workers can take first steps to achieve the following: (a) identify HT victims and perpetrators; (b) stabilize and control the HT situation; (c) prepare victims and pass information on to trained investigators; and (d) refer victims to specialized service providers [26]. Therefore, multidisciplinary teams can provide more efficacious outcomes from victimization, survivorship to restoration [14].

Although the estimates of HT may not be completely known, studies have documented that victims of both sex and labor trafficking interact with healthcare providers [7,24,27]. Literature suggests that victims seek and receive medical care at some point of their traumatic experience in bondage due to the constant abuse they experience [26,28]. Studies have shown that anywhere between 28–88% of HT victims come into contact with a healthcare provider at some point during their period of victimization [29-31]. Thus, these patient–provider interactions create a vital opportunity for intervention. These interactions can potentially facilitate trafficked patients a possible way out from their victimization and oppression. Nonetheless, the nature of victimization resulting from their trafficking creates stereotypes, stigma, and shame. Thus, these psychological processes frequently inhibit their victimization disclosure to healthcare providers [26]. Research-based training and better systems for their identification could provide a platform for supporting such vulnerable populations [28]. The intersection of healthcare with trafficked victims is a significant opportunity for identification and support. If clinicians, frontline responders, law enforcement agents and other needed professionals such as public health practitioners seek to adequately assist HT-patients in transitioning from victimization to survivorship, and reintegration to society, strategic approaches must be in place [7,15,24-26]. This is not to say that these processes are simple. These processes of patient–provider require trust, genuine care, victim-centered, and trauma-informed practices, as well as operating in a supportive institutional environment that has the resources to actually identify, assist, and support trafficked patients [6,9,14].

It is important to note that through these processes of identifying and assisting such patients, the goal is not to *rescue* nor *fix* them. On the opposite, the framework of assisting such population is to provide an *integrative* care that provides patients the time and support needed to move beyond victimization (see Discussion Section—*Compassionate Care*) [6,32]. Therefore, here lies the importance of abovementioned qualities of care, as well as best identification protocol practices. Such practices together will assist patients who are trafficked to begin seeing their value and realizing that their healthcare providers could provide the needed support for escaping their entrapment. Thus, healthcare, and social service providers must collaborate as a team to optimize care of the identified ST-patient by ensuring access to suitable resources needed for these transitions [6,33].

### 1.4. Gaps in the Literature

Despite the US being at the frontline in the anti-trafficking movement, integration of established instruments within community-based healthcare practice is slow-moving [24]. Regardless of training and curriculum development, there continues to be a gap regarding the efficacy of frontline personnel in identifying, assisting, and supporting a potential transition from victimization to survivorship [7,15,30,34]. Although an increased awareness about HT has taken place in the US, professionals that could assist victims continue to work in settings with limited use of protocols or vital community resources to increase capacity and efficacy to assist such patients [9,35]. To appropriately address the complexity of victimization of trafficked persons, to help victims exit from this coercion into survivorship, a comprehensive and multi-systemic approach are essential in response to their immediate and long-term needs [14].

### 1.5. Significance of this Research

Curriculum development, cross-training and multidisciplinary collaborative models linking disciplines including social work, the law, medicine, public health, and other service-oriented professions trained to support victims and survivors of HT is a high priority for best practices to emerge and assist this vulnerable population [7]. To create effective training, the voices of survivors of HT are essential to this process. Without incorporating their recommendations in the design, testing, implementation, and evaluation of identification protocols and best collaborative practices, the US will continue to witness inconsistencies in supporting HT-patients across multiple levels [7,27,36,37]. Therefore, building a robust literature founded on research focused on the recommendations of survivors that derive from their lived experiences when interacting with healthcare providers, is essential. Therefore, this study is one of a handful of research studies that sought to capture the recommendations articulated by a sample of survivors of ST whose insights resulted from their prior interactions with frontline healthcare providers. Consequently, these study participants’ voices provide key insights to create greater ST-patient-centered models of care and identification protocols to support their exiting into survivorship and restoration. Thus, the aim of this study was to systematically gather their recommendations with the goal of informing the future design, implementation, and evaluation of evidence-based practices in healthcare settings; especially those mostly frequented by such a population. Evaluation and research are warranted to arrive at effective standards of care within healthcare settings that are survivor-informed.

### 1.6. Theory and Framework

For this study, a theoretical framework was utilized not only to understand the complexities of the victimization resulting from ST, but also for its analysis. *Intersectionality* theory focuses on understanding the complexities of the societal structures of inequality at multiple levels in conjunction with the experiences of individuals in their everyday life [38]. Additionally, this theoretical framework posits that categories defined by society at-large; for example, race, gender, sexual orientation, socioeconomic statuses, age, among others, intersect and impact its members at the micro-level [39]. Within the context of this study, this framework is important in seeking to understand the recommendations of a cluster of survivors of ST in that their recommendations stem from their multiple social identities and lived experience. These personal experiences and traumas resulted from their daily abuse and interactions with their oppressors—traffickers and sex buyers, as well as judgmental and biased care received at the different healthcare settings, they accessed during their victimization process [26].

Therefore, seeking to understand the experiences of survivors of ST can awaken the sense of awareness and openness to the wisdom derived from their lived experience. The ability to integrate their important recommendations for better healthcare practices and assessments when serving potential ST-patients in healthcare settings can help in the formulation of much needed holistic healthcare protocols and practices. Moreover, *Intersectionality* theory is relevant to our understanding of the importance of their noted recommendations in that their experiences were contextualized by a range of interconnections of oppression including, but not limited to racism, sexism, misogyny, xenophobia, classism, heterosexism, economic discrimination, inequities, and so on. Thus, the recommendations presented in this study are a result of these junctions. Most importantly, the voices of these survivors of ST can inform daily practices for healthcare professionals and other frontline personnel in order to create a care that is grounded in the proposed framework here presented—*Compassionate Care*, along with the other principles of trauma-informed care [6,7,9,40].

Lastly, the definition of health established by the World Health Organization’s constitution is an essential blueprint when addressing the needs of vulnerable populations such as trafficked-patients and others who need specific care beyond the presented acute physical and chronic needs. This definition states that health is not only the absence of infirmity, but “is a state of complete physical, mental and social well-being” [41]. This definition along with a holistic approach to care must be applied when interpreting the findings of this study. It is essential to adhere to a holistic approach when identifying and addressing the needs of patients who have fallen victims to traffickers because they have developed multiple healthcare needs beyond the physical care [3,5,7,17,26,27,32].

## 2. Methods

### 2.1. Sampling of Participants

This qualitative exploratory study utilized an a priori sampling approach widely used in public health to gain a greater understanding about participants’ perspectives and experiences of a given subject [42]. To maximize sampling variation, data were collected across two major cities of the US. Study participants (*N* = 22) (see Table 1) were recruited via convenience sampling through victim-centered social service agencies, including survivor leaders’ organizations. These metropolitan cities constitute locations where HT activity has been documented—San Diego, CA, and Philadelphia, PA [43,44].

### 2.2. Geographic Regions of the Study—San Diego, CA, and Philadelphia, PA

San Diego, CA, is the eighth largest city in the US and is located on the coast of the Pacific Ocean, in Southern CA, immediately adjacent to the Mexican border. Therefore, its proximity to Mexico creates an even greater vulnerability for victims of HT. Compared to other states in the West and Southwest regions of the US, CA ranks as the highest state in terms of HT reported cases to the US National Human Trafficking Hotline [45]. According to the San Diego County District Attorney [46], San Diego places 13th nationally for sex trafficking of minors. Recent research confirms that San Diego has indeed a greater likelihood of documenting higher estimates of ST than previously estimated with trafficking activity reported throughout the county [43].

Although Philadelphia, PA, is not an international border city, it contains highways connecting the Northeastern and the Mid-Atlantic regions of the US. Thus, Philadelphia is considered by the anti-trafficking community advocates as a *transit state* given its easy interstate highways connections. Stakeholders who supported this project represented social service providers. In Philadelphia and surrounding metropolitan areas, these organizations included the following: Project Dawn’s Court, The Valley against Sex Trafficking (VAST), The Institute to Address the Commercial Sexual Exploitation at Villanova University Charles Widener School of Law, and the Women Organized Against Rape (WOAR). In San Diego, CA, supporting organizations consisted of Freedom from Exploitation, GenerateHope, Hidden Treasures Foundation, Soroptimist International of Vista and North County Inland, and Survivors for Solutions. These organizations were essential in sharing information about this study with their networks of survivors. If prospective participants were interested, they would then contact the researcher to set up a conveniently arranged meeting with the participant to ensure eligibility.

### 2.3. Safeguards for Identity Protection

This research was approved by Drexel University’s Institutional Review Board (IRB ID: 1602004287). Stakeholders and survivor-leaders also reviewed and approved the study’s interview guide prior to inviting members of their survivor-networks to participate in the study. All participants were provided a consent form explaining the study’s purpose and voluntary participation. To protect the identity of participants, consent was given verbally. Each participant also was able to choose their own pseudonym assigned to their data file. Additionally, each interview was assigned a unique identifier composed of letters and numbers (e.g., S345, P965, etc.). With these identity protective measures, there was no way to identify participants. Additionally, study participants provided their pseudo names at the end of the interview.

### 2.4. Eligibility Criteria

The eligibility criteria consisted of the following qualifications: (a) self-identified survivor of ST within the US; (b) visited healthcare settings during their trafficking experience; (c) were 18 years of age or older; (d) were able to read, write, and speak English; and (e) left their victimization at least six months prior to their participation in the study. The definition of *survivor* used in this study meant that all study participants were no longer being forced into commercial sexual exploitation, in the context of ST, and were emotionally ready to participate in the study.

### 2.5. Data Collection

This study’s data collection occurred between March 2016 and March 2017. Study participants were interviewed face-to-face or over the phone when necessary. Interviews were digitally recorded. Digital recordings were kept in a password-secured laptop. Semi-structured in-depth interviews lasted between 30–60 minutes. Participants also received a $30 gift certificate redeemable at local stores. At the end of the interview, participants chose their pseudo names. Participants’ pseudo names were used throughout the analysis.

### 2.6. Data Analysis

In-depth semi-structured interviews were transcribed verbatim. Transcriptions were then uploaded into a data management system—NVivo Mac version 11.4.0 (QSR International, Melbourne, Australia). The data were analyzed using an inductive thematic approach, including a five-step analysis process—reading, coding, displaying, collapsing, and interpreting [42]. Through the first steps, emerging themes were identified, and open-ended codes were also created. This permitted the enumeration of a range of themes with ample detail in each of those identified. Afterwards, this information was reduced to important points within each theme. Throughout these steps, researchers sought to focus on the meaning of what participants shared during the course of the study’s semi-structured interviews. During the last step, data analysts provided an overall interpretation of the identified themes, and how these were connected with one another. Investigators worked independently to code text files for data analysis, and collectively shared their observations about essential points of each theme and their connection with one another. Lastly, they arrived at a consensus in their interpretation of data. Interpretations of data were subsequently triangulated through revisiting some of the research participants’ responses during early phases of the data analysis. The main themes identified were as follows: (1) Red Flag identification, (2) suggestions on how to care for patients of ST in the healthcare setting, and (3) types of resources that could assist ST-patients.

## 3. Results

### 3.1. Sociodemographic Characteristics

Table 1 provides information on the demographic characteristics of the study participants. Participants in the study (*N* = 22) had an average age of 30. Half of the participants in the study selected “White” as their race or ethnic background followed by Black (22.72%), and Biracial and Latina (13.63%) respectively. Singleness was the most selected category for marital status followed by divorced (18.18%). The rest had only one participant for each category—Married, Living with Unmarried Partner, and Separated (4.54%). Survivors of sex trafficking in the study had an average of 4.7 years of victimization. Study participants mainly visited the Emergency Department (77.27%) and Community Care Clinics (72.72%) type of healthcare settings during their victimization period. Urgent Care and Mental Health type of settings followed with 31.81% and 22.72% respectively. In terms of their geographic location at the time of the study, a majority of the participants were recruited from San Diego, CA (81.81%), while the rest (18.18%) were from Philadelphia, PA, and its surrounding metropolitan areas. The following sections are based on qualitative analysis of the study, focused on the recommendations of the study participants for healthcare providers and health systems. These recommendations are based on their experiences during their victimization period and their interactions with such providers.

Participants’ recommendations for healthcare providers in this study are based on their experiences during their trafficking trauma and their visitations and interactions with them. These recommendations were organized based on three broad categories: (1) potential Red Flags; (2) suggestions on how to care for victims of HT in the healthcare setting; and (3) types of resources that could assist ST-patients (see Table 2). Recommendations from survivors of ST regarding signs of trauma included physical, psychological, emotional, and interpersonal over-controlling, arbitrary, and manipulative patterns of abuse from traffickers, traffickers’ assistants, and sex buyers. These recommendations also included ideas on how to best relate to ST-patients in the healthcare setting and what types of information to share with them during their medical visitations. Overall, study participants’ recommendations aimed to best inform identification and care practices designed not only to respond to their medical needs in a compassionate and caring manner but also to build trust, rapport, and an opportunity to instill hope in the patient-victims of ST who seek access to a healthcare setting.

### 3.2. Potential Red Flags among ST-Patients

#### 3.2.1. Physical Injuries, Medical Records, and Other Comorbidities

Participants in the study shared their reflections about multiple physical injuries and the reasons why they visited healthcare settings. These physical injuries experienced included, but were not limited to, broken bones—jaws, legs, arms, ribs, eye sockets, and so forth. Other injuries resulted from assaults such as being hit, punched, thrown from one side of the room to another, raped, or car accidents, to mention some. However, at other times, their lives were endangered when they tried to escape an abusive sex buyer. However, escaping at times results in major accidents and injuries. Study participants also spoke of their violent traffickers, trafficker assistants, or sex buyers perpetrating intentional harm to them such as hitting or throwing them out of cars. In this regard, *Redd’s* experiences typify the physical trauma experienced by many ST-patients. Additionally, her medical record adds to the evidence of her trafficking trauma by providing extensive documentation of her multiple injuries. *Redd* stated the following:

She [HCP] went back on my medical records and she was like ‘you’ve been here for a broken arm. You have been here for a lot of stuff… ’ I had told them that I was hit by a car, but I really had been thrown out of the car. [In another occasion] he [trafficker] broke my arm. That was different. When hit me in the head with the pole, I [also] went to the [same] hospital. She pulled all my medical records! So she was like ‘you sure have a lot of accidents’ (sarcastic tone). I was like, ‘well, I am clumsy’.

Several participants spoke about coming to the same healthcare clinic and the importance of reviewing medical records, though reviewing their records can be challenging due to the change of information ST-patients may provide at different points of entry. However, even so, looking at records was a highlighted recommendation by study participants. *Valery* recommends healthcare providers review their medical record, if possible, to see if there are any patterns of repeated injuries. She stated the following:

The provider should really look at the medical records. Always look at the medical records period. If it’s something similar every time, that’s a Red Flag. They [HCPs] could say like, ‘why is this always happening?’ [But] make a joke out of it. Like, ‘oh you seem to be like a punching bag’ or something like that. It is not funny [of course], but it is more like an icebreaker. They [the patient-victim] may follow back with something funny like, ‘Yah, just every few weeks’. Something like that, depending on the way it is answered, could be [a Red Flag].

In *Ann’s* case, due to her trafficker’s assault, she ended up seeking care in the ED as a result of a broken jaw. When healthcare providers left the attending room to complete some paperwork and release her from the hospital, the trafficker snatched her out of the room and both left without completing the medical services and discharge process. *Amy’s* trafficker continued the abuse after arriving to the hotel because she went to seek medical care without his permission and company. *Ann* made the following comment:

I got triaged when I was in the back, talking to the doctor. They gave me medicine to numb my [broken] jaw [injury resulting from her trafficker’s physical assault]. They did X-rays and stuff. He [trafficker] went back there [trafficker tracked Ann down through phone’s GPS and showed up at the hospital pretending to be the boyfriend]. Yeah, once the nurse left, he grabbed my arm, pulled the IV out, and said, ‘Let’s go!’ Then, he beat me up more when I went back to the hotel room because I went to the hospital without waiting on him.

*Leaf* shared about her corporal injuries resulting from an escaping attempt. The sex buyer was driving her to a different location than agreed upon, so she had to make a decision that resulted in corporal injuries, i.e., a broken leg and arm. After the accident, she sought medical attention in the ED with her trafficker. *Leaf* made the following comment:

Yeah, they [HCPs] asked me what happened, and I told them that I fell out the car. And that the car was driving and that I pulled too hard on the door and that I fell out the car.

Just as broken bones and other corporal injuries, malnutrition or other dietary chronic conditions could point to the constant abuse that patient-victims experience. Their long hours of work and the fact that at times traffickers use food deprivation to control their victims result in other comorbidities such as malnutrition or related symptoms. In *Adilyn’s* case, food deprivation was not the only trauma she suffered, *Adilyn* also experienced different forms of corporal injuries such as intentionally being hit with a car by her trafficker, and inhumane treatment by the trafficker’s assistant. *Adilyn* shared the following:

She was called the mistress [trafficker’s assistant], and she was like basically like the rule enforcer. And, if you didn’t listen, then you went to her farm. And, she did not do so nice stuff to you. You were starved and put on a chain; treated like an animal.

Other comorbidities included drug dependency and mental health issues.

#### 3.2.2. Sexual Risk Behavior, Repetitive STI Screenings, and Reproductive Care

Given the nature of their exploitation, sexual risk behaviors, such as having multiple partners, are another Red Flag for healthcare providers. Besides their sexual health risks through their commercial sex exploitation, having multiple types of screen testing throughout the year adds to the evidence of their sexual exploitation. Some study participants commented about the fact that their traffickers ensured their victims were free from STIs or HIV. Moreover, at times, abusers use the same healthcare setting to access women’s reproductive healthcare for their victims. One of the reasons for ensuring they were free of STIs or HIV was based on the fact that traffickers were worried about their own health since they also often use their victims as sex partners. In *Ann’s* case, her trafficker used her to engage in unprotected sexual activity. Her trafficker visited the same *Planned Parenthood* community throughout the year and brought several women for STI screenings, contraceptives, and abortions. Again, the repetitive pattern of visits with different patients could point to their trafficking and exploitation and this could potentially lead to the identification of ST-patients if this and other potential Red Flags are strongly pointing to their victimization. *Ann* shared the following:

Well, we would go every 3–6 months. He wouldn’t bring us all at the same time. … I got birth control. So I wouldn’t get pregnant. I had my abortions there … One time, when I went in for a STD check and it came back [positive]. He [trafficker] lost it, kind of, in the office because [the test was positive]. He was, ‘well you did it without protection. What the hell?!’ I never did anything without protection unless it was with him. But he just freaked out but got the medicine for me to get rid of it.

When asked about potential Red Flags for HCPs, *Amy* commented the following: Probably the multiple sexual partners. I mean. That was really the only thing that I had brought to their [HCP] attention.

*Beverly* shared the fact that she went so frequently to a facility that the staff would not provide her with a Pap smear test anymore. Nonetheless, she wanted to make sure she was free of STIs. She shared the following:

Frequent visitations. … You must get tested. You must do this. But because of the number of partners I had, they would tell me, ‘We are not going to give you another pap smear. You just had one’. You know? And I was like, ‘Let me get tested!’ You know? So I think frequent visitations is a good sign [of victimization].

She also added the following:

A lot of times, in the mental illness section [of the medical intake form], we disclose information to them [HCPs] in hopes of getting help for what we’re dealing with. So, obviously, you know if there’s abuse; if they speak about abuse going on; if they speak about multiple partners; if they speak about rape … I think those are Red Flags.

Although repeated screenings, multiple sex partners, rape, abortion or any other sign of sexual trauma could be an indication of trafficking, patient-victims, at times, may share the truth in a humorous manner. Whether they do it or not, the important takeaway of these participants’ experiences is to pay attention to the content shared during the intake phase of the medical visit. Sharing in an inconspicuous manner may be a way of revealing their trauma without being blunt about it. Detecting these nuances could be essential in seeking to identify ST-victimization. *Ann*, a survivor from the San Diego region shared the following interaction:

I: What kind of questions would they [HCP] ask you?

P: ‘How many partners have you been with? Do you use protection all of the time?’

I: Would you answer those questions honestly?

P: We wouldn’t give the honest answers. ‘Have you been with more than ten people in the last 6 months?’ No, ‘I have only been with one’. I wouldn’t, or some of the other girls would be like, ‘Yeah, we sleep with this many people’. One time, one of the girls told the [HCPs] how many. The [patient-victim] was like, ‘Oh, I was just joking’.

I: Did they say something to her?

P: They were like, ‘Oh, that’s a funny joke’. … That’s weird. Why would you joke about that?

Understanding that repetitive STI screenings throughout the year, multiple sex partners (when disclosed), and other risky sexual behavior such as unprotected sex, can constitute Red Flags of sex trafficking victimization, medical records at community-care clinics or other type of healthcare settings—E.D., mental health clinics, urgent care clinics and other sites offering free services, could point to patterns of sexually risky behaviors. Repetitive screenings can signify potential Red Flags of ST-victimization when combined with other above noted indicators. Lastly, taking seriously what is shared during the intake phase of the medical visit can add important information to the identification of ST-patients.

#### 3.2.3. Accompanying Person’s Control of Medical Care and Services

It was evident that more than half of the study participants experienced a medical care episode that was negatively impacted by the traffickers’ controlling strategies. Few participants were able to go by themselves or with a friend or relative to a medical setting. Furthermore, study participants often experienced the trafficker or his assistant’s manipulative and controlling strategies determining the care received or access to care. At times, the abusers’ strategies are such that they are able to convince the healthcare providers of their *goodness* and *care* for their victims. Some of these strategies included the following: showing up at the medical setting and interrupting the flow of the care. Others included pretending to be the *boyfriend*, *friend*, or some close relative. During the medical visit, traffickers or traffickers’ assistants answered questions on behalf of the ST-patient, instructed the HCP what medicines to give the victim, refused to leave the room, or selected particular locations that were *safe* for the suspect. In order to ensure the ST-patient would not escape, at times, the trafficker would wait outside the facility to keep a watch on her. In some medical service locations, the trafficker becomes a *frequent visitor* of the clinic to the point of building rapport with healthcare providers and staff members.

When *Redd* was treated at a hospital due to her broken bone injuries, her injuries had resulted from an altercation she had had with her trafficker. However, the trafficker was able to manipulate not only the healthcare providers, but also what she could share with them. She was afraid of him not only because he was an extremely violent person, but he was also her children’s caretaker at the time. When the trafficker came to the hospital, he manipulated *Redd* by reminding her not to disclose anything against him. He also created an *image* of himself to the healthcare staff and providers that he was an innocent person in spite of the fact that the HCPs were suspicious of the abuse *Redd* had experienced. He became demanding. *Redd* made the following comment:

So, I mean, he [trafficker] would come and go [to visit Redd at the hospital]. But every time he would come, he would be like, ‘remember, don’t say nothing, Daddy loves you and am sorry! OK?!’… And then, they [healthcare providers] even apologized to him. They’re like, ‘we are so sorry that we even assumed that you would hit your girlfriend’. Because when the nurse questioned him about my injuries, he said, ‘let me speak to your supervisor. Let me speak to the person in charge! You guys are trying to accuse me of hitting on my girlfriend. I love her!’

He started crying. He was like, ‘I would never hit her!’ I was just like, ‘dammed you are a good actor (laughs)’. And then, they [nurses] called main [hospital] management and they talked to him. And he was like, ‘I just don’t feel that just because my girlfriend has a lot of accidents, I don’t feel that’s my fault she is clumsy … blah, blah, blah’. So they (hospital staff) felt stupid. He made them feel dumb.

In the case of *Valery*, a survivor from the Philadelphia metropolitan area, her trafficker would instruct her what to say when interacting with HCPs. *Valery* recalled, the following:

Yeah, he would tell me like, ‘Don’t tell them anything about what you are doing. Just tell them that you are living with me. I’m your boyfriend … Just say that you got jumped and attacked, and you didn’t see the person’s face or anything.

Additionally, her trafficker would select healthcare settings with a shorter wait. She shared the following:

It can be very packed in those hospitals [Emergency Department]. He would choose what hospital I went to depending on how many people were waiting. There were times when we went to two or three different hospitals because it would be so busy. Like, if there were so many people in the waiting room, then he would go to the other one, so he didn’t have to wait as long.

At times, *Valery’s* trafficker would control the delivery of medical care by explaining what had happened to her even though the healthcare provider would ask her the questions. In other instances, he kept her from receiving the care she needed. *Valery* shared the following:

Like, he did most of the talking, but they would ask me questions um about like … You know like, “On a 1 to 5, how are you feeling?” Type of stuff. Exactly, where does it hurt? Or, what kind of pain are you feeling? They asked me what happened. I kind of give them a gist. Then, he would explain it more. Or there were times when he didn’t want me to go to the hospital. He wouldn’t let me go. He kind of told me to suck it up. He was mad because a John robbed me that time.

#### 3.2.4. Body Modifications

*Beverly* cautions healthcare providers about forming stereotypes about ST-patients of sex trafficking:

I think a lot of times, when we think about human trafficking in America, we think about Asians or Indians. Or a lot of times, we don’t think about Americans. So, it’s kind of hard because the appearances … you know … [For example] when I was in the Valley, there are a lot of people of upper-class and a lot of escorts and call-girls who are being trafficked. And, you know … they are gorgeous! They look like they got it all together, and no one knows what’s going on.

Although there is no one *look* that identifies ST-patients just as *Beverly’s* quote denotes, there are some physical signs that can begin to provide HCPs some evidence pointing to the ST-patient’s trafficking trauma. For example, tattoos may be a common trend in today’s patients’ appearance in any given healthcare setting; however, if these body modifications depict certain messages, it can potentially be another Red Flag. The tattoos that most likely would be indicative of sex trafficking often contain images or drawings that portray money signs, phrases such “give money,” and traffickers’ names. *Redd* commented the following:

If somebody is coming in there with like tattoos, and I am not saying that everybody who has tattoos is human trafficking victim, but come’n now [shows interviewer her tattoos—money sign in her forearm and points to her side of her face where she has another one], like ‘give money’ ‘money bags’ ‘dollar signs all over my body’ a dollar sign on my face … somebody’s name tattooed on my face … It’s only common sense!

#### 3.2.5. Other Signs and Caution

Study participants pointed to other potential signs as indicators of ST victimization. For example, inconsistencies with the reasons for their injuries, being too distracted, constantly looking at cell phone messages, looking around, anxious, nervous or *skittish*. Caution must be exercised regarding not stereotyping or judging patients as these potential Red Flags can overlap with other patients who are not victims of ST. If a potential ST-patient is accompanied by an older controlling person, study participants suggested it be considered as a potential Red Flag. Nonetheless, the other components of this accompanying person would include characteristics of a personality that is domineering, forceful, controls the interaction between the patient and the healthcare provider, does not want to leave the room, is much older than the patient, fills out her forms, and is holding on to the patient-victim’s identification card. Although, no one isolated sign is indicative of suspicion, having a combination of the noted different signs adds to the probability of ST. It is also important to know that at times, according to some participants, the suspect could appear to be a *nice* person, but still is controlling in the context of the decision-making process.

### 3.3. Supportive Healthcare Practices when Caring for Patient-Victims of Sex Trafficking

#### 3.3.1. Do Not Ignore Potential Signs

ST-patients due to their trauma and fear most likely are not willing to share the truth about their oppressive conditions. However, at times, ST-patients do share some *pointers* that partially speak about their trauma. *Rose* and others shared about times when they would disclose either seriously or jokingly about the number of sex partners to provide some context about their current situation. However, healthcare providers appeared to dismiss what these ST-patients shared by continuing to administer the protocol and not asking deeper questions or pausing the intake process. Study participants consistently noted that healthcare providers continued with their medical assessment and dismissed the patient-victim’s need for intervention. *Rose*, a San Diegan survivor, made the following comment when describing and providing her insights about these types of interactions,

So, the Planned Parenthood visits were for periodic checks for STDs basically. And, when I went in there, one of the questions they would ask me was, ‘How many sex partners have you had in the past 30 days?’ And, I would always say, ‘I don’t know, maybe 50?’ And then, they didn’t make any comment about it at all, and then, I’d say, ‘Oh, yeah. I’m an escort’. And, they wouldn’t say anything about it, and I thought, ‘This is such bullshit’. And then, they just basically handed me, you know, they always handed me condoms. They would literally hand me like hundreds of them.

In the case of *Beverly*, the Planned Parenthood’s healthcare providers had a similar response as in the case of *Rose*. The providers seemed to ignore the fact that she had had countless sex partners and continued to attend the same clinic across time. *Beverly* recounted the following:

When I went in to get testing [STI], even though you fill out some paperwork, I think one of the first questions on there was, “Do you know about how many partners have you been with the last 30 days?” You know? I could never recall. So I would always say, “a few hundred” or “very many”, something like that. I’m pretty sure that they had an idea, but I don’t think they knew about how to talk about it, you know what I mean? They never said anything after I would share how many partners I had.

Study participants here expressed their experience as they visited several times healthcare settings and were not asked any questions beyond the protocol. It seems that this may be a signal for potential commercial sex exploitation. At times, it may be uncomfortable for a provider to ask a patient a deeper question. However, if a provider wants to assist ST-patients during their daily interactions with them, a provider should first reflect about the ways a ST-patient shares information as an important first step to identifying potential signs of abuse. However, healthcare providers must refrain from stereotyping patients since there may be some who are not under the oppression of a trafficker. When in doubt, healthcare providers should seek trained co-worker support. A collaborative model would work best to double check Red Flags and provide feedback among providers. Identifying and assisting ST-patients should be a team effort.

#### 3.3.2. Feeling Comfortable and Safe

Given the trauma and the daily commercial sex exploitation victims of ST experience, entering and interacting with welcoming healthcare providers in a comfortable and safe setting creates the conditions for a positive relationship with HT-patients. In the case of *Beverly*, she visited many different family planning community clinics and a behavioral health clinic as well. When comparing her experiences between these two settings, she enjoyed going to the latter one mainly because it felt safe and providers were hospitable. This is what she shared:

First and foremost, it was an extremely secure location. They were [also] extremely hospitable there. If you were hungry, they would feed you. … , they had like little … sandwiches or chips or whatever, [they would] feed you. They were super hospitable. The therapist who talked to me would bring up a concern and ask me if I needed help. They would give me several resources. They were great [laughs]. So, I felt safe being in the facilities because I knew that everyone was searched. In the waiting room, they had televisions there too. When you go and you tell them you’re there for an appointment or you’d like to see so and so, they would often ask if you’d eaten. And, I don’t know if they did it just to me because I looked homeless or what. They would ask me if I’d eaten. … Stuff like that, you know? They were really friendly. I don’t know. They were nice. I felt it was. I enjoyed going there.

For *Beverly*, this was a safe environment, but also a place where the healthcare providers showed care and concern about her well-being and were ready to provide resources if she wanted them, and even food when necessary.

Other participants shared that in order to build an environment that feels comfortable and safe, it is essential to build rapport with the ST-patients. One of the key themes throughout the recommendations was the idea of encouraging healthcare providers to be more sensitive toward the ST-patient even if they do not understand completely what the patient is going through.

#### 3.3.3. Asking Questions Respectfully

Along with the idea of creating the conditions likely to make ST-patients feel more comfortable, study participants also shared the importance of providers who are willing to ask questions when there are potential signs of abuse; yet, with sensitivity and respectfully. Asking questions or requesting information from the patient must be done in a respectful and caring demeanor without showing judgment or disgust. At the same time, asking questions to simply find information without providing concrete guidance or resources suitable for the ST-patient could potentially be more detrimental when trying to build rapport and trust. It is essential that healthcare providers who identify potential Red Flags seek the advice of those already trained to serve this population. Understanding their trauma is an important first step to assist ST-patients, as well as acquiring skills to interact with them. Being empathic and caring is essential as is the ability to withhold judgment. *Alexandra*, a survivor from San Diego area shared the following:

My recommendation would be to ask questions very carefully. Many [patient-victim] may not be comfortable with talking about their situation. Try to be more careful. Try to be more caring, you know? Give them more information on where they can get help … The doctor that I was talking to asked me, ‘Well, why are you in this situation? Why are you on the streets?’ … Some people see prostitution like it’s disgusting to them. So, they could ask the question more like ‘what happened in your life that led you to this situation?’ They [HCP] could ask questions in a different way, you know? Making sure they [HCP] don’t ask them in a way that they wouldn’t want to be questioned, you know?

Just as asking questions in a caring, compassionate and respectful way while withholding judgment is important, so is the ability of the healthcare provider in seeking to build rapport, trust, and a willingness to intervene in the lives of ST-patients they may interact with during their daily practices.

#### 3.3.4. Caring about the Well-Being of the Patient-Victim

Participants spoke about the need for providers to reach out and show empathy through simple actions that demonstrate care for the ST-patient’s well-being. Study participants recommended healthcare providers to show *compassion*. Compassion can be translated in different forms. In the case of *Tiara*, compassion included a willingness to follow a protocol in place, as well as going the *extra step*. Caring about the well-being of the ST-patient, in her opinion, also encompassed not being *judgmental* or showing sarcasm towards the patient. Ultimately, the goal of caring for the well-being of the patient should reassure the ST-patient that the provider is seeking her well-being. *Tiara* shared the following:

First and foremost, I’ll let them know [healthcare providers] to develop a compassionate view on the patient. To not be so judgmental and quick to judge based off a certain situation. [Also] to be willing to reach out and give that person help. The help and care that that person needs. To just be patient with the victim. You know. Ask questions. And, just to reassure [with emphasis] the victim that everything is okay.

Providing care that reflects *compassion* towards the ST-patient is important given that it has the potential to instill a sense of hope as it is being shared. This compassionate view, will not only diminish a judgmental view, but it could assist healthcare providers to build trust with the patient. Reaching out and assisting ST-patients seem to be essential for creating a positive relationship between the patient-victim and provider. *Jazzy* added the following:

Just kind of like make the person [patient-victim] feel like you are very compassionate and very concerned about her health.

On the other hand, many participants experienced the opposite of *Compassionate Care* when they sought care. For *Leaf*, a survivor of Philadelphia, PA, she had to experience *almost death* experiences due to the violence of her sex buyers and traffickers before exiting her victimization. However, during her visits to healthcare settings, unfortunately, she recounted that she never felt compassion from the healthcare providers. Nonetheless, *Leaf* believed it is an important component of caring for ST-patients. She explained that for a person who is constantly living in the *grip of survival*, meeting someone who cares for their well-being could have a positive impact in their lives. *Leaf* recounted the following:

I never had anybody (in the healthcare setting) say to me, ‘this is a great resource for you’ or ‘you should talk to somebody’, or ‘if you need help, these are some people who can help you’. Something so simple. It doesn’t have to be, you know, ‘I want to hear your whole story’. It can be something subtle and simple. And to just let her know that someone *cares*. Someone’s thinking about her. When people said to me (on the streets), ‘I’m going to pray for you’, that was enough for me because you are in the grip of survival. And for you to hear someone say that to you, even if you don’t know the person, shows that they care. Yet, nobody took the initiative to actually *really care* for me [in the healthcare setting].

It may be as simple as practicing active listening and sharing encouraging words with her, something that she may never have heard in a healthcare setting.

Lastly, the participants raised the notion of understanding personal assumptions of the “Other.” According to some study participants, when there is a lack of understanding from the healthcare provider as the reasons for why ST-patients ended up in this entrapment, it will limit healthcare providers’ assessment ability. As a consequence, it will also limit their ability to build rapport and trust in their relationship, which are important components for intervention.

#### 3.3.5. Understand Assumptions or Perceptions of Victimization

All human beings carry assumptions of the *Other*—who they are, where they are from, why they are the way they are, and so forth. However, lacking an awareness of these assumptions can be detrimental to building rapport and showing care for others; especially vulnerable populations who enter the healthcare setting. When study participants interacted with their healthcare providers in different cities across the West and East Coasts of the US, they reported that at times they felt judged by their providers. However, many times, these assessments may stem from healthcare providers’ preconceived notions and/or personal biases. Their perceptions of the reasons why ST-patients entered their current victimization or why they remain in such abusive circumstances has the potential to impact treatment regimens. *Rose*, a San Diegan survivor, explained the following:

A lot of people who don’t really have any experience with it [sex trafficking] and they kind of buy into the idea of the *bad girl attitude*—’Oh, she’s just a bad girl. She just got herself mixed up in something. Or, she’s making her choice. She’s made her bed, and now, she has to lie in it. These are her choices that she’s making’. And so, they put the responsibility on the woman. Then, they kind of leave it up to you [the patient-victim] to ask for help. Whether they are not aware or ignorant, they have wrong perceptions about it … After I went to Planned Parenthood and I shared what I was doing, they didn’t even blink, you know? I kind of got belligerent with the medical community. Just seeing like, ‘They didn’t give a shit’. They didn’t ask if I needed anything, nothing.

Just as *Rose* shared, if providers’ biases and assumptions of victimization of ST-patients of sex trafficking may blind them in their ability to provide care, it is imperative to establish protocols that extend beyond administering questions to understand not only their victimization, how to identify potential Red Flags, how to ask questions, but also how to acknowledge and understand one’s personal biases and assumptions of the *Other*.

### 3.4. Resources and Information for Patient-Victims of Sex Trafficking

Understanding the types of changes that healthcare settings must operationalize in order to create the above-noted recommendations shared by study participants is crucial if healthcare settings are committed to creating an environment conducive to clinical interventions responsive to conditions resulting from trafficking victimization. Creating practices that are founded on compassionate and patient-centered care are essential when serving the needs of said patients. The following subsection contains practical ideas designed to foster changes at the institutional level to improve provider–patient rapport and build trust. Moreover, this section seeks to promote creative recommendations from study participants of how to best connect with ST-patients in the healthcare setting, as well as the steps for designing, packaging and showcasing information about essential resources that have the potential to meet a range of needs for ST-patients.

#### 3.4.1. Information—Displayed or Hidden

Several study participants recommended the provision of information about resources that they could utilize in the short- and long-term, including an 800 number to call, local resources such as housing information, hotline, and so forth. However, they noted that providing a pamphlet would not be useful. The format of this shared-information matters. The reason for their hesitancy on pamphlets was based on safety. If the trafficker were to find out a pamphlet, the ST-patient would either potentially be in danger or he would simply throw it away and the opportunity for this patient to return to the healthcare setting, most likely, would no longer exist. The emphasis, therefore, must be focused on the way this information needs to be displayed or distributed to the ST-patient. Moreover, participants had two suggestions regarding the *formats* to best present information to said patients. One format was based on a poster or display that provided visible information in the lobby of healthcare settings, bathrooms, or examining rooms. *Ann* stated the following:

You know how they have posters in the [examining] room? Have like a poster that you can look at [while you are waiting for the doctor to come in]. Not where you have to actually pick up a paper and bring it with you, but something that you can see and remember a number. You know how they have the baby’ progressing along [while they mother is pregnant]? A poster like that would be useful.

*Beverly* commented the following:

Yeah, I think I’m extremely resourceful. So, yeah, when I’m waiting, I do a lot of reading. So, having the resources on display is extremely helpful because then, in confidentiality, I can decide whether or not if I want to utilize the information.

The other format emphasized focused on providing information that the ST-patient could carry with her without raising the suspicion of the trafficker. Therefore, the format had to be discrete and designed in something small that she could hide in an inconspicuous manner. For example, a participant suggested printing an 800 number inside a condom’s wrapper. *Beverly* continued, saying the following:

For healthcare providers to share what is available to victims such as [name of local organizations], If the person [patient] is stand-offish, they [healthcare provider] can just say, ‘Well, I just have some information if you might be interested, and you can find it here’. Or, they give us condoms. They can have the info right inside the condoms. You know? When we leave, they [healthcare providers] give us like 20 condoms. … They can just slide it in, and you know those have numbers to inform us of what’s out there.

Others shared about the items they received by volunteers conducting outreach while they were still victims of ST while working on the streets. One of the participants was gifted what appeared to be a “lip gloss” but inside it had the 800 number of the organization she could contact. However, others shared about asking the ST-patient about saving the phone number in her cell phone contact list under a *different name*. In this way, the trafficker would not know or suspect anything about the given phone number. Nonetheless, whatever is given to the patient in physical form must be presented in a thoughtful format, to minimize her risk of suffering further violence by her trafficker. Ultimately, information must be provided in a way that will not jeopardize the rapport built (if any) and her safety.

#### 3.4.2. Other Type of Resources

Other study participants noted other important resources that could be added to plans designed to assist ST-patients. These recommendations included adding a peer-to-peer counseling team composed of survivors that could assist once the healthcare provider identified the Red Flags. A team of peer-to-peer counselors would not only allow survivors to be involved in this process of intervention, but they may be more apt to instill a sense of hope that the patient-victim has found a safe haven in the healthcare setting. *Jazzy* commented, “You want to leave her with a feeling that you can help them in the future.”

Another suggestion was based on the idea of implementing training for healthcare providers including organizing panels, workshops, or Grand Rounds where survivors are part of the training. *Ann* made the following comment:

Organizing educational trainings for the nurses or the head of the ER, such workshops and panels where they get together, learn and listen to what people [survivors] have to say and experts like [named director of her previous program].

Others also suggested implementing a safety plan, with the goal of protecting the ST-patient, as well as the healthcare-setting personnel, in instances when the potential for undue harm exists. A safety plan would include not only having immediate access to community resources to address the ST-patient’s basic needs such as housing or access to food, but also confidentially, while protecting her from the trafficker’s strategic plans to get her back. This plan may also include a *word-coded* or a *color-coded* system to assist in identifying ST-patients to ensure that the trafficker cannot find her, and that he cannot enter the hospital in case the patient is hospitalized for several days. Such systems would allow for better protection of the ST-patient’s confidentiality, while making her whereabouts unknown to her trafficker. Although these systems may require more intricate planning and complexity, the goal is to keep the ST-patient safe from the trafficker’s hands. *Valery* explained the following:

Um, having a code word and all that. Also having a specific list of people that can call and show up at the hospital. And if you aren’t on that list, unless you know that code word, you aren’t getting in. You just can’t. … To me the most important thing is safety. [However], you have to be trained to know the process, already have that in place because if you do it on the whim, it’s not going to work. You got to have some type of plan [and be ready for different situations]. Yeah, just in case because you never know what … can happen at any time. She can have just jumped out of the car and ran into the hospital trying to get away from him. And he might be hunting her down, but as long as there is a plan in place, it can work.

*Valery* also explained that sharing useful information for ST-patients to address their needs is important as they may begin to see other possibilities outside their victimization; especially if they are seeking to attempt an escape from their victimization. *Valery* shared the following:

Some girls might be ready and might not know they’re ready. For instance, like they might just don’t want to be in the situation, but they feel like that’s it. That’s life. So, you just want to, kind of probe them and figure out what they do in life. If you find out that’s what she’s doing and she specifically tells you, tell her like, ‘Look, if you don’t want to be in that lifestyle and you want to get some help, we can help you. Probably find you a bed some place, and they can help you eventually find an apartment and get a job. All that stuff’.

The above-noted recommendations point to two main themes—safety, and the importance of ensuring a type of care that goes the *extra mile*. If the patient-victim can start to feel respected, cared for, and that she can trust healthcare providers because they have shown her that she is respected both as a patient and human being, she might begin to see a way out of her trafficking victimization. Moreover, these recommendations potentially lay the groundwork for a successful intervention because actions that demonstrate caring, safety, and respect can have a restorative effect on the ST-patient’s human dignity. It is perhaps the integration of these elements that collectively offers greater potential for finally eradicating an ST-patient’s oppression.

## 4. Discussion

The findings of this study indicate that despite the immense challenges to identify potential ST-patients in the healthcare setting, there are potential Red Flags that could be observed and acknowledged by healthcare providers in the course of their daily responsibilities. The results of this study also confirm other researchers’ findings focused on the importance of compiling possible *Red Flags* among ST-patients [6,27,47-49]. Most importantly, this study’s findings are based on the survivors’ voices and their experiences during their trafficking victimization period. It is important to note that study participants caution healthcare providers to be aware of their personal biases and from stereotyping potential ST-patients. Someone could be trapped in this type of atrocity; however, the Red Flags may not be so visible.

In terms of *supportive practices* that healthcare settings and providers need to explore and potentially adopt, this study points to important recommendations specific to attitudes related to the personal treatment of the ST-patient, creating a safe and comfortable environment, and practicing a type of care that adds to extant frameworks including *trauma-informed* and *victim-centered* care—a *Compassionate Care*. Additionally, withholding judgment about the ST-patient, demonstrating respect when taking a medical history and/or asking probing questions, and being aware of one’s personal biases must be reflected in the institutions’ culture, as well as in the healthcare providers’ daily practices. Moreover, these practices always warrant being subsumed within both *trauma-informed* and *victim-centered* approaches to care, as well as being part of the healthcare providers’ medical ethos [25,27,37,50,51]. Nonetheless, this study introduces the concept of *Compassionate Care* in the context of caring for ST-patients. To the authors’ understanding, this concept has yet to be identified as a finding in previous literature in the context of HT. *Compassionate Care* outside of the HT literature is defined as a type of care mainly provided by healthcare professionals, characterized by recognizing, understanding, and empathizing with the patient’s concerns suffering or pain. However, it does not stop there. The ability to empathize with the patient can move the healthcare provider to positive actions that typically lead to ameliorating the situation of their patient. Thus, healthy provider-ST-patient partnerships can potentially promote autonomy and self-efficacy in both providers as well as in their patients. This *Compassionate Care* approach to care seems to offer the potential for additional positive outcomes in the context of the provider–patient relationship, as well as the patients’ health outcomes [52]. This approach and applied skill to the care of ST-patients has the potential to facilitate a breakthrough in their interaction, assessment of needs, and provision of suitable resources for paving a successful exit from their oppression. A *Compassionate Care* approach could also be applied to other vulnerable patients that frequent healthcare settings. In other words, it is not limited to only ST-patients, but any other vulnerable population that visits the healthcare setting and whose needs may overlap with trafficked victims.

For example, since *Compassionate Care* shows that the provider is willing to go the extra *mile* to ensure the ST-patient knows the provider cares for her, it could encourage them to perceive healthcare and the healthcare setting as a potential advocate and safe haven, respectively. If identified ST-patients could begin to trust the healthcare provider, there might be a window of opportunity to instill hope in their mind. This hope could lead the victim to be encouraged about receiving support and to leave her perpetrator. The recommendations of survivors are not only intended to better identify ST-patients in the healthcare setting, but also to identify key elements necessary to build trust between the patient and the provider. Without *Compassionate Care*, the identification of and opportunity to assist victims of sex trafficking in the healthcare setting is limited.

Once the healthcare provider has gained efficacy in learning how to identify Red Flags, and types of extant resources, information provided to such patients is just as essential as using the framework of compassionate care. This study highlights the importance of having a well-developed plan and knowledge of suitable local and national resources and partners needed to support this population. If the ST-patient is ready to leave her trafficker, the healthcare provider and the healthcare team must be ready to respond to her immediate needs as well. In cases where ST-patients are willing to share their status and are ready to leave their current situation, this study found a great need for a plan that ensures immediate protection and integrated support. If a protocol or plan is not in place, there are limited opportunities to assist ST-patients further. It is understandable that resources for patients who have been identified as victims of ST can be challenging to find; especially in this current strained healthcare system of the US. This is why a *collaborative* model to assist and address the needs of ST-patients is essential in anti-trafficking efforts, as earlier stated [6,10,25,26].

Additionally, types of information displayed, and its formatting are essential as well. Having posters displayed at the healthcare setting for everyone to see not only shows institutional awareness of the problem, but may simultaneously encourage this population to begin trusting this particular healthcare setting. Having resource-rich information disseminated to the ST-patients that is hidden from perpetrators is also important for their safety and the future opportunities for their return to the same medical setting. These recommendations point to the imperative of prioritizing the eradication of HT by implementing creative ways to address their condition. ST-patients live in constant danger under the oppression of their traffickers. The ultimate goal, therefore, is to keep her safe, build rapport and trust in the context of patient–provider interaction, and to offer opportunities for future intervention so that she can exit her victimization successfully.

Lastly, if a trauma-informed, victim-centered and *Compassionate Care* framework can be implemented, it would be fitting for healthcare settings to set up a peer-to-peer counseling system. Through this system, identified victims could be further assisted by peers whom they could find trustworthy given their similar victimization experience. This would not only reflect a victim-centered approach to care, but it will also allow for the integration of fellow survivors in the future intervention of other ST-patients. This approach to care seems to result in a two-fold outcome. First, it seeks to facilitate a greater prospect for building trust and rapport with the ST-patient. Second, it could also empower other survivors as they assist in the process of intervention. Showing ST-patients that the healthcare provider is someone who cares and is willing to go the *extra mile* by listening to the ST-patient with empathy offers the potential to build rapport, trust, and provide a more integrated process through the framework of *Compassionate Care*. These are vital components for assisting and supporting such a population in healthcare settings, including similar interactions with other frontline personnel.

### 4.1. Future Recommendations

Future research and evaluation are needed on how to better incorporate the HT signs, language and symbols into healthcare settings that can lead to inclusion, safety, and better outcomes for diverse HT survivors with multiple social identities. It is important to examine whether and how the content is physically displayed in and disseminated by healthcare facilities in a way that reflects diverse racial, ethnic, gender, and sexual orientation identities of all those who are victims of HT. More insights are needed about the effectiveness of these best practices.

Moreover, incorporating and evaluating training that includes the understanding of victims’ psychological trauma, as well as traffickers’ strategies for controlling their victims, are critical to creating trauma-informed and victim-centered care systems. They are essential not only to identify potential victims, but also to improve current intervention efforts. Most importantly, evaluation of medical care that is framed within the context of *Compassionate Care* towards the ST-patient could provide insights about the impact it might have on ending victims’ oppression and life-threatening circumstances of abuse and violence.

Future research could also gather more recommendations from survivors in other non-clinic-based health settings such as within the mental health and behavioral healthcare systems. In the current study, only one survivor spoke about a mental health system, but survivors do interact with social workers in other systems who could serve as first responders as well. Furthermore, findings from this study indicate a need to increase the systematic ways to track ST-patients in the healthcare setting into a national database. A national database would assist given the migratory nature of some trafficking rings where victims of ST are moved from state to state. Overall, more research is needed to inform best practices for the early identification and detection of HT; specifically, when applying the framework of *Compassionate Care* to this vulnerable population.

### 4.2. Limitations and Strengths

This study relied on a convenience, non-random sampling technique for recruitment of participants, which consequently limits the generalizability of its findings to the nation’s population. Thus, findings derived from this study may not represent the experiences identified by individuals across other regions of the US, or those residing in non-metropolitan areas. Similarly, the same limitations emerge relative to victims of HT who are foreign nationals and residing in the US. Because this study was derived from a small sample, generalizability is therefore limited, especially given extant contextual and environmental considerations. The conclusions derived from this study, however, relied on the lived experiences of a cluster of female US-born victims of HT, and their prior interactions with healthcare providers in various types of healthcare settings within two of the nation’s top 10 largest metropolitan areas. Nonetheless, small and localized samples are generally considered a hallmark of qualitative research, which confers advantages such as the ability to iteratively respond to emergent themes and concepts as the research evolves, and the ability to provide textured narrative description yielding insight into processes, meanings, and social dynamics [53].

Another limitation in this study could be recall bias. The study’s participants relied on their ability recalling events during prior interactions with healthcare providers which could potentially lead to inaccuracies, including recall bias of detailed information about their prior victimization. However, securing their participation from two geographically diverse regions of the US, and incorporating two types of data sets, qualitative and quantitative, enhances the potential to increase the validity of the study’s findings. Moreover, having designed and tested a semi-structured interview guide prior to initiating this study, comprising questions that were clear to the study participants in conjunction with relying on an experienced and well-trained interviewer, would also appear to reduce the potential for recall bias.

Additionally, similar to other research about sensitive topics when working with a hidden and vulnerable population, considerations of social desirability bias may be applicable. Some study participants might have minimized their responses of what they perceived to be undesirable behaviors or attitudes when questioned by their healthcare providers. However, given the high amount of saturation on the topic achieved in the current analysis, we judge these threats to be minimal.

## 5. Conclusions

This study sought to understand the significance of the narratives and views of a cluster of ST survivors, including their recommendations for healthcare providers who are uniquely positioned to identify and intervene on their behalf while delivering medical care to them. Unfortunately, their perspectives are frequently absent in the research literature, despite the fact that this hidden population often experiences persistent trauma, stigma, marginalization, racism, and sexism, among many other forms of oppression. This study identified recommendations that can inform healthcare providers’ critical decision-making processes in identifying, intervening, and providing suitable resources on behalf of ST-patients to move beyond victimization. These findings suggest a need for implementing specific education on how to identify potential Red Flags; understand personal assumptions, stigma, and biases about and toward ST-patients, and suggestions for supportive practices in order to best care for such a population in the healthcare system. These recommendations from survivors of ST aim to build rapport and trust, thus assisting in the process of exiting their victimization by offering suitable trauma-informed and patient-centered approaches of care. Specific resources that can be useful for ST-patients include the following: (a) peer-counseling, (b) displayed posters in the lobby or waiting rooms, and (c) discrete information given during their clinical appointment, without the knowledge of the trafficker. Thus, healthcare providers can play a positive role by creating physical environments and survivor-informed practices and protocols that are welcoming, provide for their safety and build trust and rapport with potential ST-patients.

## Figures and Tables

**Table 1.** Socio-demographic Characteristics.

Socio-Demographic Characteristics	*N* = 22 (%)
**Age**	
Mean	30
**Race**	
White	11 (50.00)
Black	5 (22.72)
Biracial	3 (13.63)
Latina	3 (13.63)
**Marital Status**	
Single	15 (68.18)
Divorced	4 (18.18)
Married	1 (4.54)
Living with unmarried partner	1 (4.54)
Separated	1 (4.54)
**Years of Sex Trafficking Victimization** *	
Mean	4.7
**Healthcare Settings Visited During Victimization**	
Emergency Departments	17 (77.27)
Community Care Clinics (Planned Parenthood)	16 (72.72)
Urgent Care	7 (31.81)
Mental Health	5 (22.72)
**Geographic Location**	
San Diego, California (CA)	18 (81.81)
Philadelphia, Pennsylvania (PA)	4 (18.18)

* Two participants were victims for less than a year. They were given the value of 0.5 when calculating the mean.

**Table 2.** Recommendations of sex trafficking survivors for healthcare providers.

Potential Red Flags Among ST-Patients	
**Physical Injuries, Medical Records, and Other Comorbidities**	The provider should really look at the medical records … If it’s something similar every time, that’s a Red Flag.
**Sexual Risk Behavior, Repetitive STIs Screenings, and Reproductive Care**	Probably the multiple sexual partners. I mean. That was really the only thing that I had brought to their [HCP] attention.
**Accompanying Person’s Control of Medical Care and Services**	Like, he did most of the talking, but they would ask me questions um about like … You know like, “On a 1 to 5, how are you feeling?” Type of stuff.
**Body Modifications**	If somebody is coming in there with like tattoos … [shows interviewer her tattoos—money sign in her forearm and points to her side of her face where she has another one], … somebody’s name tattooed on my face …
Supportive Healthcare Practices when Caring for Patient-Victims of Sex Trafficking	
**Do Not Ignore Potential Signs**	… Planned Parenthood … one of the questions they would ask me was, ‘How many sex partners have you had in the past 30 days?’… I don’t know, maybe 50?’ … I’d say, ‘Oh, yeah. I’m an escort’.
**Feeling Comfortable and Safe**	[I]t was an extremely secure location … They were super hospitable. The therapist who talked to me would bring up a concern and ask me if I needed help. They would give me several resources. … So, I felt safe being in the facilities because I knew that everyone was searched …
**Asking Questions with a Caring Approach**	My recommendation would be to ask questions very carefully … Many may not be comfortable with talking about their situation … Try to be more caring, you know? Give them more information on where they can get help …
***Compassionate Care*—Reaching Out and Caring**	I’ll let them know [healthcare providers] to develop a compassionate view on the patient. To not be so judgmental and quick to judge based off a certain situation. [Also] to be willing to reach out and give that person help … To just be patient with the victim. You know. Ask questions … reassure the victim that everything is okay.
**Understand Assumptions or Perceptions of Victimization**	After I went to Planned Parenthood and I shared what I was doing, they didn’t even blink, you know? I kind of got belligerent with the medical community. Just seeing like, ‘They didn’t give a shit’. They didn’t ask if I needed anything, nothing.
Shared Information with Patient-Victims of Sex Trafficking at the Healthcare Setting	
**Information—Displayed or Hidden**	Have like a poster that you can look at, while you are waiting for the doctor to come in … something that you can see and remember—a number. You know how they have the baby’ progressing along while the mother is pregnant?

ST-patient, sex trafficking patient.

## Data Availability

The data presented in this study are available on request from the corresponding author. The data are not publicly available due to privacy of participants.
